# Validity and repeatability of three commercially available in-shoe pressure measurement systems

**DOI:** 10.1186/1757-1146-7-S1-A67

**Published:** 2014-04-08

**Authors:** Carina Price, Daniel Parker, Christopher J Nester

**Affiliations:** 1 Centre for Health Science Research, University of Salford, Greater Manchester, M6 6PU, UK

## Background

In-shoe pressure measurement devices are commonly used in research and clinical settings to quantify pressure on the plantar foot. Various in-shoe pressure measurement devices are currently available and they differ in their size, number of sensors, sensor type and therefore their loading response and accuracy. Previous comparisons focus on pressure plates [1]. An in-shoe study highlighted that the F-Scan system became erroneous at pressures over 200kPa and the repeatability of the Novel device was high [2]. However the long loading durations (11 minutes) studied has limited application to a real-life setting. The validity and repeatability of each system effects their appropriateness for applications within clinical and research test settings. This abstract, therefore aims to establish the suitability of each device to test protocols with differing loading magnitudes and durations.

## Methods

**Figure 1:**
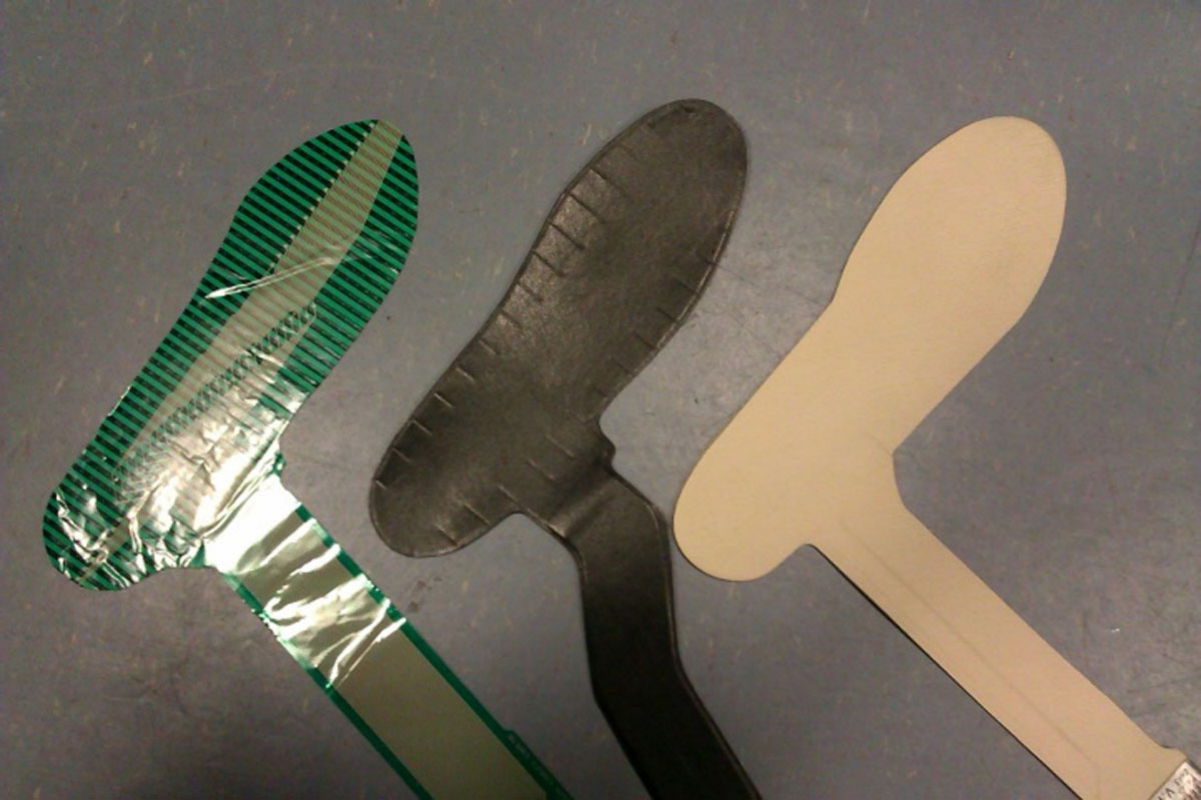
Test insoles in size 4 from left to right: Tekscan, Medilogic, and Pedar.

Three in-shoe pressure measurement devices (Medilogic, Tekscan and Pedar, Figure 1) were examined for their repeatability and validity in a 2 day x 3 repeated trial design. The testing procedure was undertaken in the Novel calibration device (TruBlue) applying an even load over the entire insole surface for UK 4 and 10 insoles. The protocol applied a range of pressures (50, 100, 200, 300, 400, 500 and 600 kPa) for 0-30 seconds. The repeatability (ICC) and validity (RMSE) of the held load (for 0, 2, 10 and 30 seconds) were outcome variables.

## Results

The Pedar system displayed low overall RMSE (3.5 kPa) for all magnitudes and durations applied and a peak value of 7.5 kPa when measured at 600 kPa for 30 seconds. The Tekscan (31.5 kPa) and Medilogic (27.3 kPa) systems RMSE was substantially higher, with maximum RMSE values of 58.4 and 50.4 respectively. The between-day repeatability of the measured pressure values varied between systems. Medilogic ICC values ranged from .334-.947 at 100 and 600 kPa respectively with a mean of .667. Pedar ICC values ranged from .345-.917 kPa at 300 and 600 kPa respectively with a mean of .638. Tekscan ICC values ranged from .042-.919 at 50 and 500 kPa respectively with a mean of .614, after exclusion of the 600 kPa data. All insole systems produced the highest ICC values for pressure values above 100 kPa.

## Conclusions

The choice of an appropriate pressure measurement device must be based on the, duration of loading, magnitude of loading and the outcome variables sought. Medilogic and Tekscan are most effective between 200-300 kPa; Pedar performed well across all pressures.
